# Regulation of mitochondrial activity controls the duration of skeletal muscle regeneration in response to injury

**DOI:** 10.1038/s41598-019-48703-2

**Published:** 2019-08-22

**Authors:** Laurence Pessemesse, Lionel Tintignac, Emilie Blanchet, Fabienne Cortade, Elodie Jublanc, Remi Demangel, Guillaume Py, Chamroeun Sar, Gérard Cabello, Chantal Wrutniak-Cabello, François Casas

**Affiliations:** 1 INRA, UMR866 Dynamique Musculaire et Métabolisme, 2 place Viala, Université Montpellier, F-34060 Montpellier, France; 2 INSERM, UMR1051 Institut des Neurosciences de Montpellier, Université Montpellier, F-34091 Montpellier, France; 3 Neuromuscular Research Center, Departments of Neurology and Biomedicine, Pharmazentrum, University of Basel, 4056 Basel, Switzerland

**Keywords:** Ageing, Trauma

## Abstract

Thyroid hormone is a major regulator of skeletal muscle development and repair, and also a key regulator of mitochondrial activity. We have previously identified a 43 kDa truncated form of the nuclear T3 receptor TRα1 (p43) which stimulates mitochondrial activity and regulates skeletal muscle features. However, its role in skeletal muscle regeneration remains to be addressed. To this end, we performed acute muscle injury induced by cardiotoxin in mouse tibialis in two mouse models where p43 is overexpressed in or depleted from skeletal muscle. The measurement of muscle fiber size distribution at different time point (up to 70 days) upon injury lead us to unravel requirement of the p43 signaling pathway for satellite cells dependent muscle regeneration; strongly delayed in the absence of p43; whereas the overexpression of the receptor enhances of the regeneration process. In addition, we found that satellite cells derived from p43-Tg mice display higher proliferation rates when cultured *in vitro* when compared to control myoblasts, whereas p43−/− satellites shows reduced proliferation capacity. These finding strongly support that p43 plays an important role *in vivo* by controling the duration of skeletal muscle regeneration after acute injury, possibly through the regulation of mitochondrial activity and myoblasts proliferation.

## Introduction

In skeletal muscle, satellite cell regeneration is a crucial process in the daily life of normal population to maintain muscle performance after injury as well as during ageing to limit muscle strength decline. Upon muscle injury, the skeletal muscle stem cells (thereafter referred as satellite cells) which are normally quiescent are activated. Once activated, the satellite cells start to proliferate, migrate to the site of injury and subsequently differentiate and fuse to form newly multinucleated fibers to repair and rebuild the damaged myofibers^1^.

Thyroid hormone (TH) is a major regulator of muscle development and metabolism. It stimulates muscle growth by increasing the number and diameter of muscle fibers^2,3^ and regulates the contractile features of adult muscle fibers^4^. TH is also a key regulator of mitochondrial activity. These functions of TH involve both TH receptor α (TRα) and β (TRβ). Recently, the role of TH^5–7^ and TRs^8^ have been investigated in the context of injury induced muscle regeneration. Notably, satellite cell-specific deletion of the 5-deiodinase 3 gene (D3), the enzyme that inactivates T3, severely impairs skeletal muscle regeneration^5^. In addition, TH Transporters MCT8 and OATP1C1 were recently shown to be required to ensure a normal skeletal muscle regeneration^7^. Similarly, the introduction of a dominant-negative knock-in mutation of the TRα gene (TRα1PV) in mice results in an impaired regeneration of skeletal muscle whereas a similar mutation in TRβ gene (TRβ1PV) has no effect^8^. To conclude the authors suggested that the local control of T3 and TRα plays an essential role during *in vivo* skeletal muscle regeneration. These studies strongly suggest that TRα gene mediates the effects of T3 during skeletal muscle regeneration.

However, the putative involvement of p43 during muscle tissue repair after acute injury was not considered. P43 is a 43 Kda truncated form of the nuclear receptor TRα1 which stimulates mitochondrial activity^9–11^. We showed that p43 overexpression stimulates mitochondrial activity and potentiates terminal differentiation in myoblasts, whereas the inhibition of this pathway induces the reverse changes through the control of myogenin, c-Myc, and calcineurin expression^12–14^. In addition, p43 overexpression in these cells induces a prominent increase in type 1 fibers^14^. More importantly, we demonstrated *in vivo* that the specific overexpression of p43 in skeletal muscle (p43-Tg) increases mitochondrial respiration and induces a shift in the metabolic and contractile features of muscle fibers toward a slower and more oxidative phenotype^15^. Conversely, p43 depletion in mice (p43−/−) reduces mitochondrial respiratory chain activities and induces a shift toward a faster and more glycolytic muscle fiber phenotype^16^. In addition, whereas the absence of p43 leads to an increase of muscle mass^16^, its overexpression induces muscle atrophy during aging^17^. These sets of data indicate that p43 regulates muscle mass as well as the metabolic and contractile properties of myofibers through the modulation of mitochondrial activity. However, the involvement of p43 in muscle regeneration process remains to be addressed.

Using mouse models overexpressing p43 in skeletal muscle or lacking p43, we report here that p43 plays an important role *in vivo* by controling the duration of skeletal muscle regeneration after acute injury. Upon acute injury, skeletal muscle regeneration is strongly delayed in the absence of p43, whereas the overexpression of the receptor enhances of the regeneration process. Moreover, we found that satellite cells derived from p43-Tg mice proliferated faster compared to control myoblasts, whereas satellites cells providing from p43−/− proliferated slower. Thus indicates that p43 controls myoblasts proliferation through the regulation of mitochondrial activity.

## Methods

### Animals and ethics statement

Male mice were housed and maintained on a 12-hour light/dark cycle (lights on at 7:00 pm). Food (A04, SAFE) and water were provided *ad libitum*. All animal experiments were performed according to European directives (86/609/CEE and 2010/63/CEE) and approved by the Comité d'Ethique en Matière d'Expérimentation Animale: Région Languedoc-Roussillon (CEEA-LR-12091). Our institution guidelines for the care and use of laboratory animals were observed. Our animal facility is approved by the Departmental Veterinary Services (No. C34-172-10) and our Ministry of Research (No. 4962). P43−/− mice, lacking specifically the mitochondrial T3 receptor p43 and p43-Tg mice were generated in our team as described previously^15,18^. All mice used in these studies were back-crossed more than ten times into the C57BL/6 background. We generated our colony by crossing p43−/− mice with WT C57BL/6J breeders, and generated future generations of WT controls. According to the European Directive 2010-63-EU, mice were observed daily for the general health status and mortality scoring. Any obvious signs of disease, injury and behavioral disorder indicating pain were recorded. If signs persist for more than 48 hours the animal was euthanized by cervical dislocation.

### Skeletal muscle injury

6-month-old male mice were used to study skeletal muscle regeneration. After anesthesia by Isofluorane, 25 µl of cardiotoxin (10 µM in saline) (C9759, Sigma-Aldrich) was injected in the right tibialis anterior muscle to induce muscle injury. The contralateral (uninjected) left tibialis anterior muscle was used as control. Mice were euthanized 4, 10, 28 and 70 days afterward and the tibialis muscles were collected. For each genotype (WT, p43−/− and p43-Tg) and for each group (4, 10, 28 and 70 days), 6 mice were used.

### Histological studies

After euthanasia by cervical dislocation, Tibilais anterior muscles were collected, freshly frozen in Tissue-Tek (Microm Microtech), and then stored at −80 °C. Ten µm sections were stained with hematoxylin-eosin or Sirius Red. For immunostaining the sections were fixed in PBS, 4% PFA at room temperature (RT) 5 min, permeabilized 30 min in PBS, 20% horse serum, 0.1% triton at RT, and incubated with the primary antibody in a solution of PBS, 20% goat serum, 1% BSA, 24 h at 4 °C. Sections were washed in PBS 3 × 10 min and incubated with the secondary antibody in PBS 1 hour at 37 °C. Sections were washed in PBS, 2 × 10 min, incubated 30 seconds with DAPI and washed once in PBS 10 min and mounted.

For the morphometric analysis, muscle sections were scanned using a NanoZoomer (Hamamatsu Photonics, Japan) with a 20X objective. Definiens developer 7.1. software was used to analyze and quantify the pictures for each entire area.

### Antibodies

Primary antibody used: anti-Laminin (1/200, rabbit polyclonal, Sigma L9393); anti-Troponin T (T6277, Sigma, diluted at 1:50); anti-Myogenin (sc-576, Santa Cruz Biotechnology, diluted at 1:400); anti-MyHC isoforms (M7523, Sigma, diluted at 1:1000); and anti-α-Tubulin (DM1A, Cell Signaling, diluted at 1:6000).

### Isolation of satellite cell-derived myoblasts and cell culture

Primary myoblasts were derived from mice between 4 and 6 weeks of age. Concisely, murine myoblasts were isolated from hind limb muscles after enzymatic digestion by pronase. Cells were plated at a density of 20,000 cells/cm^2^ on Matrigel®-coated Petri dishes (BD Biosciences) in Ham’s F10 supplemented with 20% horse serum and 1% penicillin/streptomycin. Two days after, cells were washed with Ham’s F10 (Gibco) and placed in complete growth medium supplemented with 5 ng/ml basic fibroblast growth factor (bFGF, Invitrogen), 20% horse serum and 1% penicillin/streptomycin (Growth Medium, GM). The myoblast population was enriched by differential adhesion compared with fibroblasts by serial 30-min preplate procedures after trypsinization. To induce differentiation (0 h of differentiation), primary myoblasts were reached at 80% confluence and switched in Differentiation Medium (DM) consisting of Ham’s F10 containing 15% horse serum and 1% penicillin/streptomycin.

Cell proliferation was measured by total DNA quantification using a QuantiFluor dsDNA system (Promega) according to the manufacturer’s protocol.

Cytoimmunofluorescence myoblast differentiation was assessed by observation of morphological changes and accumulation of muscle-specific marker. After methanol fixation and three washes with PBS-gelatine (0.2%), cells were labelled with an antibody raised against Troponin T (T6277, Sigma, diluted at 1:50). Nuclei were stained with Hoechst 33258 (1 μg/ml). To assess the extent of differentiation, the fusion index (percentage of nuclei incorporated into myotubes relative to the total number of nuclei) was calculated 72 h after adding the differentiation medium using ImageJ software.

### Protein levels

Protein levels were assessed by Western blotting. Total proteins were lysed in Tris-NP40 (100 mM Tris, 0.7% NP40, pH 7.4) and measured using the Bio-Rad protein assay. 20 μg of protein were electrophoresed onto 10% SDS-PAGE gels and blotted onto nitrocellulose membrane. Membranes were probed with antibodies raised against Myogenin (sc-576, Santa Cruz Biotechnology, diluted at 1:400), all MyHC isoforms (M7523, Sigma, diluted at 1:1000) and α-Tubulin (DM1A, Cell Signaling, diluted at 1:6000). Signals were revealed using a Clarity^TM^ Western ECL Substrate kit (Bio-Rad), and proteins were visualized by enhanced chemiluminescence using the ChemiDoc Touch Imaging System (Bio-Rad) and quantified with Image Lab™ Touch Software (version 5.2.1).

### Statistical analyses

All results are presented as means ± sem, or as percentages. Statistical significances of the differences between groups were evaluated with Student’s t-test.

## Results

### Modulation of p43 signaling impairs early muscle regeneration

To determine whether p43 can modulate regeneration after acute injury, we injected cardiotoxin (CTX) into the TA muscle of WT, p43−/− and p43-Tg mice. In these mice, under basal conditions, as previously described, we found that quadriceps, gastrocnemius or tibialis weights were increased in the absence of p43 and conversely decreased in mice overexpressing p43 when compared to controls (Tintignac *et al*., submitted). We as well confirmed our previous observations^15–17^ showing that the specific overexpression of p43 in skeletal muscle (quadriceps) increases mitochondrial respiratory chain activities (Complex I and IV) whereas a depletion of this receptor leads to a reduction of these activities when compared to WT mice.

**Figure 1:**
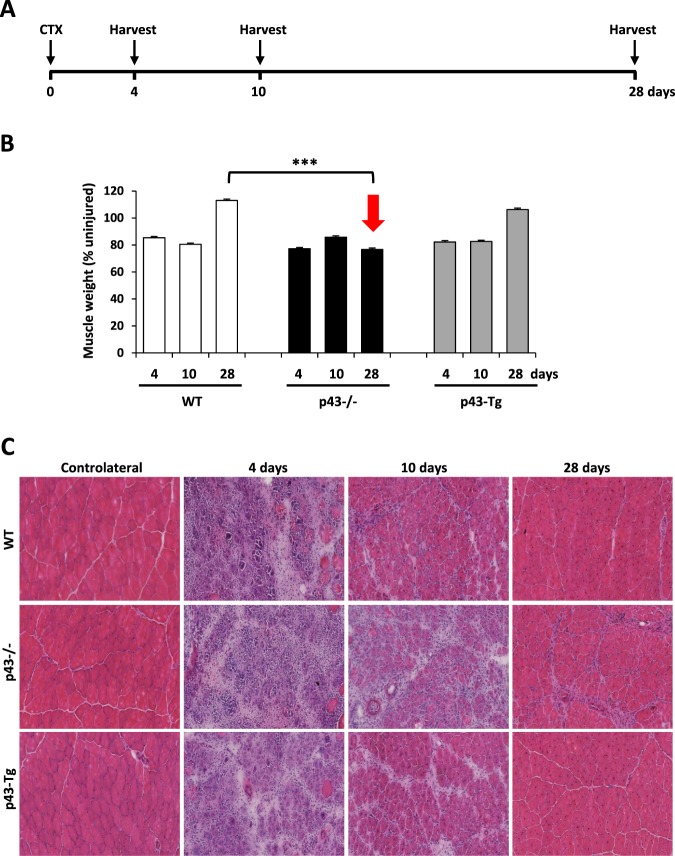
Modulation of p43 signaling impairs early muscle regeneration at 28 days post injury. (**A**) Experimental setup. Tibialis muscle were injured by a single CTX injection and analyzed at different times following injury. (**B**) Assessment of muscle recovery evaluated by the ratio between the injected and contralateral tibialis weight analyzed at different times following injury (n = 6 for each group). (**C**) H&E staining on cryosections of uninjured and regenerating muscle tissue at 4, 10 and 28 d.p.i in wild-type, p43−/− and p43-Tg mice. Statistical significance: ***p < 0.001, Student’s *t*-test. Results are expressed as ± sem.

After acute injury, contralateral and injured tibialis were collected, weighted and analyzed at different time points during regeneration (Fig. 1A). Assessment of muscle recovery was evaluated by the ratio between injected versus contralateral tibialis weight. We observed that after injury, and for all genotypes, the tibialis weight ratio was initially decreased (day 4 and day 10), before increasing at day 28 post injection in both WT and p43-Tg whereas this ratio remained low in p43−/− tibialis suggesting an impairment of muscle regeneration in these mice (Fig. 1B). We next performed Hematoxylin and Eosin staining on tibialis muscle sections. At 4 d.p.i, histological analysis of regenerating muscles from all genotypes showed early regenerating myotubes and numerous mononucleated cells (likely proliferating myoblasts and infiltrating immune cells). At 10 d.p.i, regenerating control muscles were already composed of newly formed myofibers with centrally located nuclei, whereas p43−/− muscles contained a higher amount of fibrotic tissue and inflammatory cells, and smaller myofibers. At 28 d.p.i, control muscle had fully regenerated and muscle architecture was restored with normal myofiber, whereas p43−/− muscles displayed aberrant myofiber morphology and again, a huge amount of fibrotic tissue (Fig. 1C).

**Figure 2:**
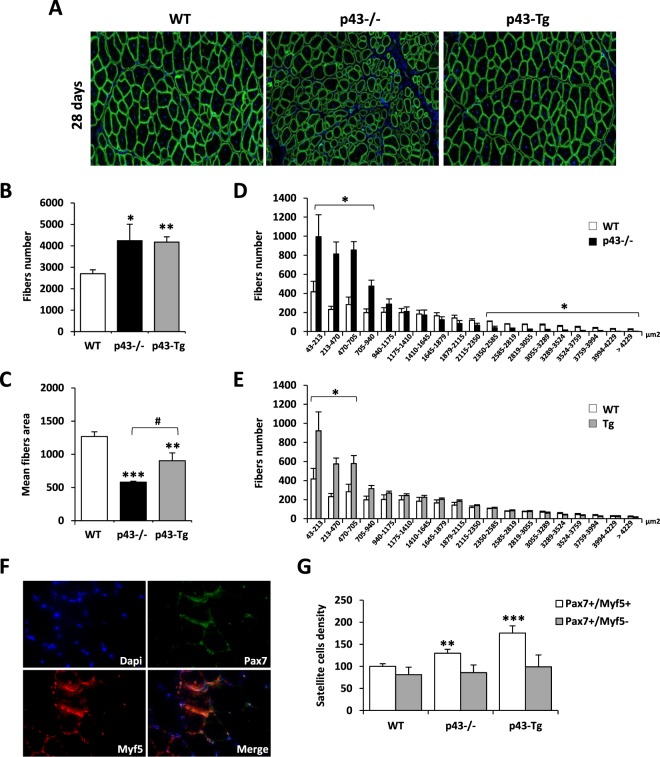
Influence of the modulation of p43 signaling on muscle characteristics at 28 days post injury. (**A**) Anti-Laminin staining on cryosections of regenerated Tibialis muscles in wild-type, p43−/− and p43-Tg mice at 28 d.p.i. (**B**) Total fibers number in wild-type, p43−/− and p43-Tg mice at 28 d.p.i. (n = 6 for each group). (**C**) Mean fibers area in wild-type, p43−/− and p43-Tg mice at 28 d.p.i. (n = 6 for each group). (**D**) Fiber size distribution in wild-type and p43−/− mice at 28 d.p.i. (n = 6 for each group). (**E**) Fiber size distribution in wild-type and p43-Tg mice at 28 d.p.i. (n = 6 for each group). Statistical significance: *p < 0.05; **p < 0.01; ***p < 0.001. Student’s *t*-test. Results are expressed as ± sem. (**F**) Example of Anti-myf5, Anti-Pax7, Dapi and merge staining on cryosections of Tibialis muscles performed in wild-type, p43−/− and p43-Tg mice. Arrows indicate the nuclei stained by immunolabeling. (**G**) Quantification of the Pax7+/Myf5− and Pax7+/Myf5+ cells density based on staining in (**F**) in wild-type, p43−/− and p43-Tg mice.

In order to further measure the regenerative capacity of each genotype (WT, p43−/− and p43-Tg mice), we performed immunostaining for Laminin to visualize the basal lamina at 28 d.p.i. We first observed that p43−/− sections display a higher myofiber density (per field) with abnormal morphologies when compared with controls (Fig. 2A). When assessing the number of myofiber, their cross-sectional area (CSA) and their size distribution, we found that the total number of myofibers per tibialis muscle was strongly increased in both p43−/− and p43-Tg mice when compared with controls (Fig. 2B), whereas their median fiber CSA was strongly decreased in particular in absence of p43 (Fig. 2C). Quantification of myofiber and measurement of their CSA in uninjuried tibialis (Fig. S1) showed that regenerated myofibers are increased and smaller in all genotypes. Representation of myofiber size distribution shows that the proportion of small myofibers was drastically increased in p43−/− and p43-Tg mice compared with control mice (Fig. 2D,E), whereas the number of myofibers with a higher CSA fall in p43−/− injured tibialis (Fig. 2D). Taking in account these observations suggesting that the initial number of satellite cells pool could be different in each genotype, we further quantified the different subpopulations of satellite cells expressing Pax7, with or without Myf5 in basal condition in uninjured tibialis. We found no difference between the genotypes in the density of the Pax7+/Myf5− cells whereas the Pax7+/Myf5+ cells pool is increased in both p43−/− and p43-Tg mice when compared with controls tibialis muscles (Fig. 2F,G).

### Modulation of p43 signaling delays muscle regeneration

**Figure 3:**
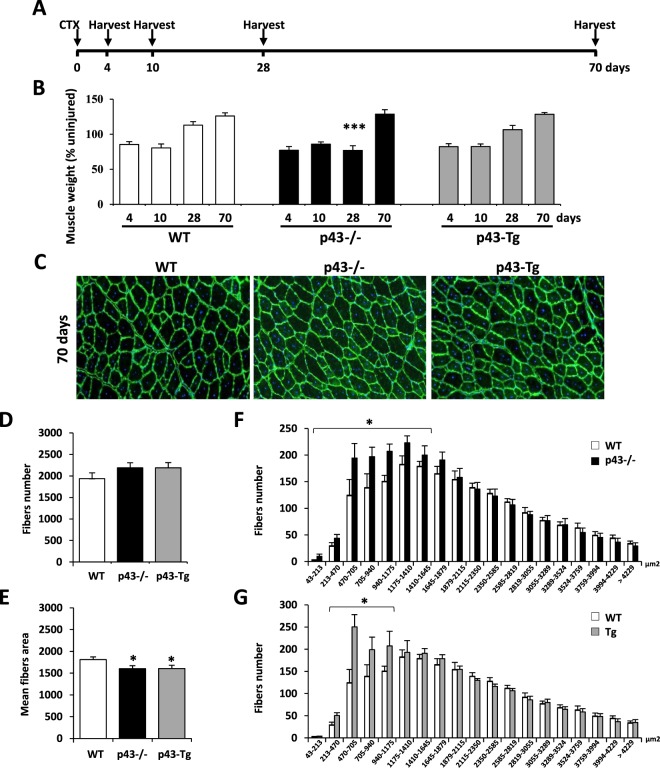
Modulation of p43 signaling delays muscle regeneration. (**A**) Experimental setup. Tibialis muscle were injured by a single CTX injection and analyzed at different times following injury. (**B**) Assessment of muscle recovery evaluated by the ratio between the injected and contralateral tibialis weight analyzed at different times following injury (n = 6 for each group). (**C**) Anti-Laminin staining on cryosections of regenerated Tibialis muscles in wild-type, p43−/− and p43-Tg mice at 70 d.p.i. (**D**) Total fibers number in wild-type, p43−/− and p43-Tg mice at 70 d.p.i. (n = 6 for each group). (**E**) Mean fibers area in wild-type, p43−/− and p43-Tg mice at 70 d.p.i. (n = 6 for each group). (**F**) Fiber size distribution in wild-type and p43−/− mice at 70 d.p.i. (n = 6 for each group). (**G**) Fiber size distribution in wild-type and p43-Tg mice at 70 d.p.i. (n = 6 for each group). Statistical significance: *p < 0.05; ***p < 0.001. Student’s *t*-test. Results are expressed as ± sem.

**Figure 4:**
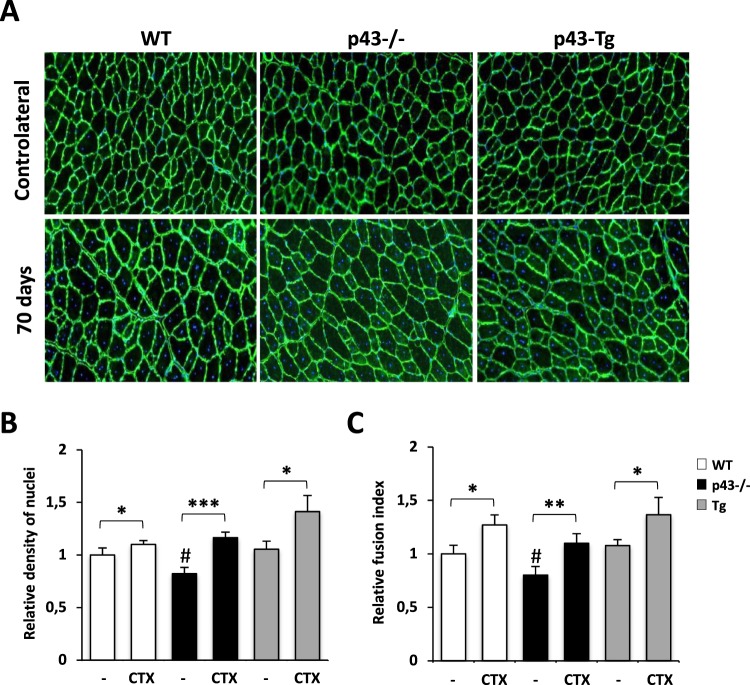
Measurement of muscle characteristics after injection of Cardiotoxin at 70 d.p.i. (**A**) Anti-Laminin staining on cryosections of contralateral tibialis and regenerated Tibialis muscles at 70 d.p.i in wild-type, p43−/− and p43-Tg mice. (**B**) Relative density of nuclei in contralateral tibialis or after injection of Cardiotoxin in wild-type, p43−/− and p43-Tg mice (n = 6 for each group). (**C**) Relative fusion index in contralateral tibialis or after injection of Cardiotoxin in wild-type, p43−/− and p43-Tg mice (n = 6 for each group). Statistical Significance: *p < 0.05; **p < 0.01; ***p < 0.001, Student’s *t*-test. Results are expressed as ± sem.

The observation that in p43−/− mice 28 d.p.i, the muscle architecture was abnormal with numerous small and rounded shaped myofibers lead us to hypothesize that the regeneration process might be incomplete rather that deficient in these mice. In order to address this point, we further characterized tibialis muscle at day 70 after CTX injection (Fig. 3A). Surprisingly, we found that the weight ratio between the injected and contralateral tibialis was increased for all genotypes, strongly indicating that a compensatory growth event arises in p43−/− mice at late time point after injury (70 dpi) (Fig. 3B). Histological analysis achieved by Laminin staining revealed that the architecture of the tibialis muscle was fully restored with normal myofibers for all genotypes even if a small proportion of centrally located nuclei were still present at 70 d.p.i. (Fig. 3C). As previously described, we further determined the number of myofiber, their cross-sectional area (CSA) and their distribution. At 70 d.p.i. all genotypes display the same number of myofiber despite a small but significant reduction of the mean fiber area in p43−/− and p43-Tg mice when compared with control mice (Fig. 3D,E). Interestingly, the measured values, at 70 dpi and for all genotypes were quite similar to those measured in contralateral tibialis (Fig. S1) indicating that at this stage the regenerative process was near to be complete. Analysis of the fiber size distribution demonstrated that the overall pattern was very similar between the genotypes excepting for small myofibers that remained higher in p43−/− and p43-Tg mice when compared with control mice (Fig. 3F,G). Next, we compared the density of nuclei (number of nuclei by cross section area) and the fusion index (number of nuclei by fiber) between the injected and contralateral tibialis (Fig. 4A–C). Uninjured tibialis display no significant differences between WT and p43-Tg animals whereas the density of nuclei and the fusion index were decreased when measured in p43−/− muscle (Fig. 4B). At 70 d.p.i, the density of nuclei and the fusion index in the tibialis muscle where found increased for all genotypes when compared to contralateral uninjured muscle (Fig. 4C).

### Modulation of fibrosis p43−/− mice

**Figure 5:**
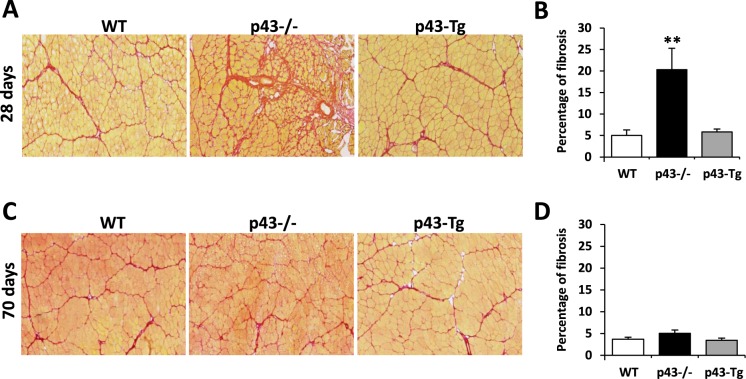
Modulation of p43 signaling affects early muscle fibrosis. (**A**,**C**) Sirius Red staining on cryosections of regenerated Tibialis muscles in wild-type, p43−/− and p43-Tg mice at 28 d.p.i and 70.d.p.i. (**B**,**D**) Quantification of Sirius Red staining (H) muscles in wild-type, p43−/− and p43-Tg mice at 28 d.p.i. and 70.d.p.i. (n = 6 for each group). Statistical significance: *p < 0.05; **p < 0.01; ***p < 0.001. Student’s *t*-test. Results are expressed as ± sem.

Growing evidence indicates that both fibrotic and myogenic regenerative phases might overlap within the injured skeletal muscle in early time after injury^19^. In order to address this point, we performed Sirius Red staining on injured tibialis at early and late d.p.i. Interestingly, we found a strong increase of fibrosis in p43−/− mice at 28 d.p.i when compared to control or p43-Tg animals (Fig. 5A,B). When observed at 70 dpi, the amount and the distribution of fibrotic tissue are equal through-out all genotypes (Fig. 5C,D). These observations revealed that p43−/− injured muscle display an acute phase of fibrosis resorption between 28 and 70 d.p.i. and finally exibit the same pattern of fibrotic tissue distribution as observed in WT and p 43-Tg injured muscle.

### p43 regulates myoblasts proliferation

**Figure 6:**
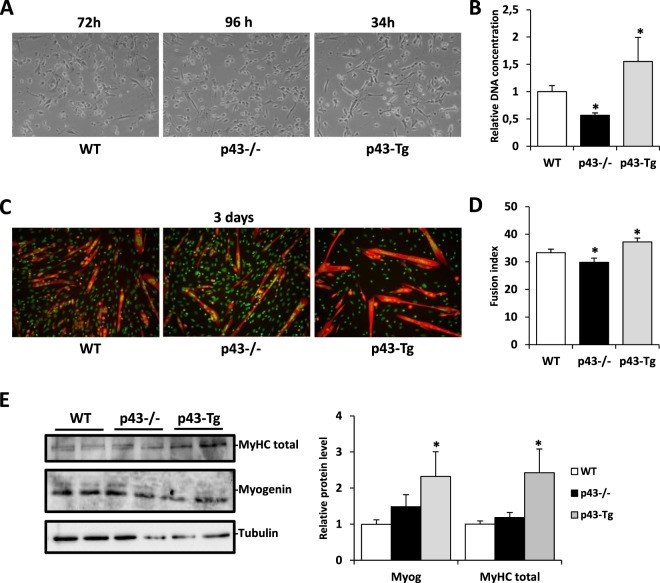
p43 regulates myoblasts proliferation. (**A**) Time needed to reach 80% of confluence in p43−/−, p43-Tg and WT primary myoblasts. (**B**) Proliferation rate monitored at 34 hours attested by relative DNA concentration in p43−/−, p43-Tg and WT primary myoblasts using Quantifluor (n = 5 for each group). (**C**) Cytoimmunofluorescence studies using antibodies raised against Troponin T were performed on WT, p43−/− and p43-Tg myoblats after 3 days in differentiation medium. (**D**) To assess the extent of differentiation, the fusion index (percentage of nuclei incorporated into myotubes relative to the total number of nuclei) was calculated. (**E**) Myogenin and total MyHC protein levels were analysed by Western blot (n = 6 for each group). Typical blots are shown. Proteins were quantified with Image Lab™ Touch Software. Statistical significance: *p < 0.05. Student’s *t*-test. Results are expressed as ± sem.

We have previously shown that p43 overexpression stimulates mitochondrial activity, cell proliferation and consequently potentiates terminal differentiation of avian and/or murine myoblasts, whereas direct inhibition of this pathway induces the reverse changes^12–14^. As expected, similar results were obtained in satellite cells derived from p43−/−, p43-Tg and WT mice. First, WT primary myoblasts required 72 hours to reach 80% confluency after plating, p43 overexpressing cells only needed 34 hours while 96 hours were in p43 deficent cells (Fig. 6A). In addition, the proliferation rate monitored at 34 hours showed that compare to WT myoblasts, p43-Tg myoblasts proliferated faster and p43−/− myoblast slower (Fig. 6B). Then, we tested the ability of these cultured myoblasts to differentiate in myotubes (Fig. 6C). After 3 days of differentiation we found that p43 overexpression induced a moderate increase of myoblast fusion index compared to controls (fusion index: 33,3% in WT vs 37,2% in p43-Tg, P < 0.05) whereas the absence of p43 led to a slight decrease (fusion index: 33,3% in WT vs 29.8% in p43-Tg, P < 0.05) (Fig. 6D). In addition, p43 overexpression induced a significant increase of total MyHC and Myogenin protein levels (Myogenin: 1 in WT vs 2,32 in p43-Tg, P < 0.05; MyHC total: 1 in WT vs 2,42 in p43-Tg, P < 0.05) whereas no difference was observed in p43−/− myoblasts compared to WT myoblasts (Fig. 6E).

## Discussion

Taking advantage of our genetic models we investigated here the role of p43 in skeletal muscle in response to muscle injury. First, we observed that 28 days after acute injury, muscle regeneration is affected by the absence of p43 as shown by the decreased tibialis muscle weight. In addition, despite the histological analysis indicates a strong increase in the number of myofibers, their size remains very small and their morphology abnormal when compared to WT tibialis. In p43−/− mice, 70 days are required to fully recover skeletal muscle similar to that of control animals. On another hand, at 28 days after acute injury, the tibialis muscle weight of mice overexpressing of p43 is already similar to the contralateral tibialis muscle. In addition, histological analysis indicates a strong increase in the number of small myofibers whereas the distribution of bigger fibers did not change. Here again, 70 days were required to fully recover skeletal muscle.

Moreover we further provide evidence that p43 mediates in part the influence of T3 during muscle tissue repair after acute injury. In addition, we bring evidence that p43 ensures the proper duration of skeletal muscle regeneration. However, its absence does not compromise the programs required for satellite cell lineage progression and skeletal muscle regeneration. These differences may be attributed to the different time point we selected for the post-injury analysis; only 14 days after cardiotoxin injection for the study of Milanesi^8^ and co-workers; 40 days in the report addressing the role of the D3 gene^5^; whereas we found that 70 days post-injury were necessary to obtain an almost complete regeneration in absence of p43.

Because skeletal muscle maintenance in adult and/or after injury rely on the number of satellite cells found in initial pool, we quantified in tibialis muscle the subpopulations of resident satellite cells expressing Pax7, with or without Myf5. Pax7+/Myf5− cells are destined for self-renewal whereas Pax7+/Myf5+ cells are committed satellite cells which can ensure fiber growth and repair. Interestingly, we observed that only the Pax7+/Myf5+ cells pool is increased in both p43−/− and p43-Tg mice when compared with controls tibialis muscles. Thus result explains why the proportion of small myofibers was drastically increased in p43−/− and p43-Tg mice compared with control mice. Such an increase in absence of p43 is surprising and contradictory to the observation that satellite cells are strongly reduced in TRα1PV mice tibialis^8^. However, taking in account our previous results showing that reduction of mitochondrial activity severely decreased myoblast proliferation^12–14^, we could postulate that this increase of satellite cells pool is more likely an adaptive response to ensure skeletal muscle maintenance during adult life in p43−/− mice.

**Figure 7:**
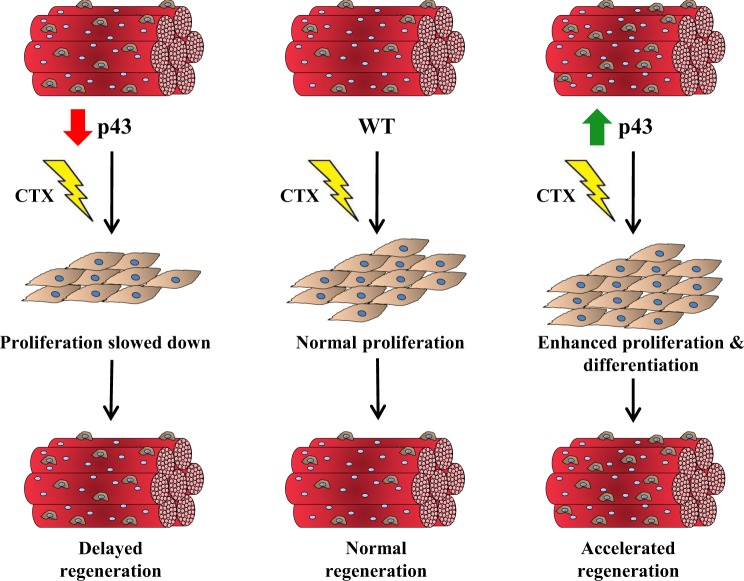
Schematic representation of p43 influence during skeletal muscle regeneration.

We have previously shown that p43 overexpression impinge on mouse and avian myoblast commitment^12–14^. Furthermore, we established that this phenotype is mediated via the regulation of the expression of the cellular oncogene c-Myc, which controls irreversible myoblasts withdrawal from the cell cycle^12^. In this study, we observed that in the quadriceps of mice overexpressing p43 specifically in muscle, the mitochondrial respiratory chain activities are increased (Complex I and IV) whereas a depletion of this receptor leads to a reduction of these activities when compared to WT mice. In addition, we found that satellite cells derived from p43-Tg mice proliferated faster compared to WT myoblasts, whereas satellites cells providing from p43−/− proliferated slower. In addition, after 3 days of differentiation we observed that p43 overexpression induced a moderate increase of myoblast fusion index compared to controls whereas the absence of p43 led to a slight decrease. Altogether these observations strongly indicate that an alteration of mitochondrial activity interferes with the ability of satallites cells to proliferate. We hypothesize that p43−/− satellite cells proliferate less efficiently when compared to WT cells, leading to an increased duration of the proliferation period and a delayed muscle regeneration. Conversely, p43 overexpression stimulates proliferation and might pushes prematurely the satellite cells toward differentiation. However, this precocious induction of terminal myoblast differentiation induced by p43 is to the detriment of an optimal myoblast proliferation period (Fig. 7).

Acute muscle injury frequently results in muscle fibrosis, reminiscent of a deposition of collagen and other extracellular matrix proteins produced by muscle fibroblasts but also by fibro/adipogenic progenitors and skeletal muscle progenitors. The interaction between satellite cells and these cells from the connective tissue is necessary to produce increased levels of extracellular matrix components to ensure an efficient and effective muscle repair, suggesting that both fibrotic phase and myogenic regeneration are overlapping^19^. In physiological conditions, the components of the extracellular matrix are degraded as regeneration and growth of new myofibers proceeds whereas the persistence of fibrosis reflects a defective muscle repair as seen in numerous muscular diseases. In p43−/− mice we observed an increase of fibrosis 28 days after cardiotoxin injection which is no longer present at 70 days. Considering that the myogenic process is inevitably coupled with fibrotic process^20^, the accumulation of extracellular matrix components at 28 days post injury points toward the involvement of the marked delay in muscle regeneration in this mouse model rather than being pathologic.

In summary, we found that p43 plays an important role *in vivo* by controling the duration of skeletal muscle regeneration after acute injury. Our findings indicate that after injury, p43 signaling, through the regulation of mitochondrial activity, plays an essential role in the control of myoblasts proliferation. Our findings brings a significant contribution to our understanding of satellite cells biology and muscle regeneration, and demonstrate that a tight control of endogenous p43 in muscle stem cells is required for their function within the skeletal muscle tissue. In addition, our data provide evidence that p43 mediates in part the influence of thyroid hormone during muscle tissue repair after acute injury. Our results open new perspectives for the development of therapeutic strategies envisioning that the local control of mitochondrial activity could restore the impaired myogenesis seen in muscle disorders and during ageing.

## Supplementary information

supplementary information

## Data Availability

The datasets generated during and/or analysed during the current study are available from the corresponding author on reasonable request.
