# Supplementary material for: Regulation of mitochondrial activity controls the duration of skeletal muscle regeneration in response to injury
Source: Sci Rep. 2019 Aug 22;9:12249. doi: 10.1038/s41598-019-48703-2 (PMC6706433; doi:10.1038/s41598-019-48703-2)
Supplement: Supplementary file 1 — supplementary information [file 41598_2019_48703_MOESM1_ESM.docx]

**Regulation of mitochondrial activity controls the duration of skeletal muscle regeneration in response to injury**

Laurence Pessemesse^1^, Lionel Tintignac^3,1^, Emilie Blanchet^1^, Fabienne Cortade^1^, Elodie Jublanc^1^, Remi Demangel^1^, Guillaume Py^1^, Chamroeun Sar^2^**,** Gérard Cabello^1^, Chantal Wrutniak-Cabello^1^ and François Casas^1^

^1^INRA, UMR866 Dynamique Musculaire et Métabolisme, 2 place Viala, F-34060 Montpellier, France ; Université Montpellier, F-34060 Montpellier, France.

^2^INSERM, UMR1051 Institut des Neurosciences de Montpellier, F-34091 Montpellier, France; Université Montpellier, F-34060 Montpellier, France.

^3^Neuromuscular Research Center, Departments of Neurology and Biomedicine, Pharmazentrum, University of Basel, 4056 Basel, Switzerland.

Correspondence to: François Casas, INRA, UMR866 Dynamique Musculaire et Métabolisme, 2 place Viala, F-34060 Montpellier, France ; Université Montpellier, F-34060 Montpellier, France. Email: [**francois.casas@inra.fr**](mailto:francois.casas@inra.fr)


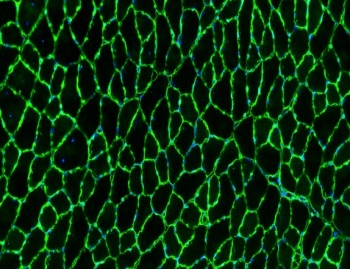

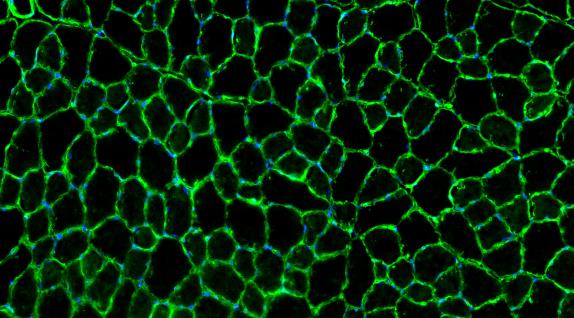

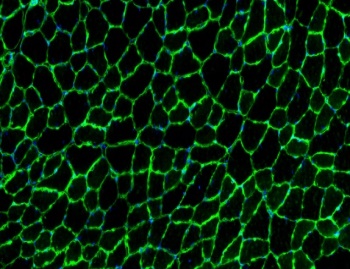


**WT**

**p43-/-**

**p43-Tg**

**A**


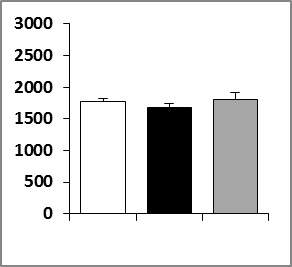

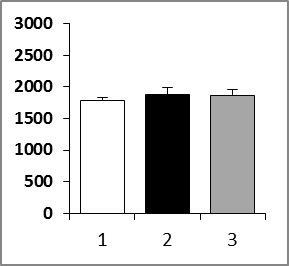


**Fibers number**

**WT p43-/- p43-Tg**

**WT p43-/- p43-Tg**

**Mean fibers area**

**B**

**C**


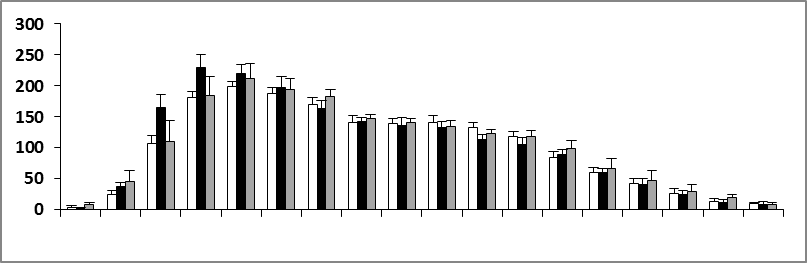


**Fibers number**

*****

*****

**WT**

**p43-/-**

**Tg**

**D**

**213-470**

**470-705**

**705-940**

**940-1175**

**1175-1410**

**1410-1645**

**1645-1879**

**1879-2115**

**2115-2350**

**2350-2585**

**2585-2819**

**2819-3055**

**3289-3524**

**3055-3289**

**3524-3759**

**3759-3994**

**> 4229**

**3994-4229**

**43-213**

**µm2**

**Supplemental figure 1.** Influence of the modulation of p43 signaling on muscle characteristics

(A) Anti-Laminin staining on cryosections of contralateral Tibialis muscles in wild-type, p43-/- and p43-Tg mice.

(B) Total fibers number of contralateral Tibialis muscles in wild-type, p43-/- and p43-Tg mice. (n=6 for each group).

(C) Mean fibers area of contralateral Tibialis muscles in wild-type, p43-/- and p43-Tg mice. (n=6 for each group).

(D) Fiber size distribution of contralateral Tibialis muscles in wild-type, p43-/- and p43-Tg. (n=6 for each group).

Statistical significance: *p < 0.05. Student’s *t*-test. Results are expressed as ±sem.

**MW WT KO Tg WT WT KO KO Tg Tg**


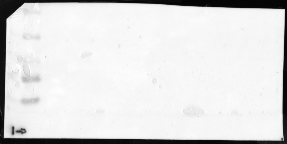


**72**

**52**

**42**

**34**


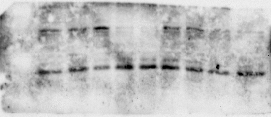


**72**

**52**

**42**

**34**

**myogenin**

**MW WT KO Tg WT WT KO KO Tg Tg**


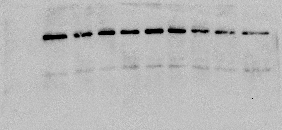


**72**

**52**

**42**

**34**

**α-tubulin**

**MW WT KO Tg WT WT KO KO Tg Tg**


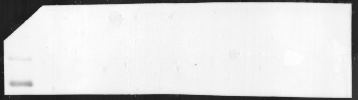


**260**

**135**

**95**


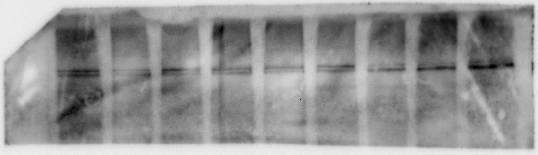


**MW WT KO Tg WT WT KO KO Tg Tg**

**MyHC**


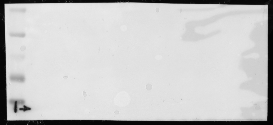


**72**

**52**

**42**

**34**


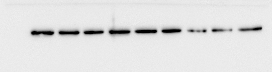


**α-tubulin**

**MW WT KO Tg WT WT KO KO Tg Tg**

**MW WT KO Tg WT WT KO KO Tg Tg**

**MW WT KO Tg WT WT KO KO Tg Tg**

**260**

**135**

**95**

**72**

**52**

**E**


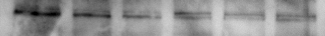


**-MyHC total**


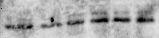


**-Myogenin**


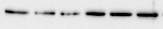


**-Tubulin**

**WT p43-/- p43-Tg**

**Figure 6.** p43 regulates myoblasts proliferation

Myogenin and total MyHC protein levels were analysed by Western blot
